# Selective Impact of HIV Disease Progression on the Innate Immune System in the Human Female Reproductive Tract

**DOI:** 10.1371/journal.pone.0038100

**Published:** 2012-06-04

**Authors:** Timothy Lahey, Mimi Ghosh, John V. Fahey, Zheng Shen, Lucy R. Mukura, Yan Song, Susan Cu-Uvin, Kenneth H. Mayer, Peter F. Wright, John C. Kappes, Christina Ochsenbauer, Charles R. Wira

**Affiliations:** 1 Department of Medicine, Geisel School of Medicine at Dartmouth, Lebanon, New Hampshire, United States of America; 2 Department of Physiology and Neurobiology, Geisel School of Medicine at Dartmouth, Lebanon, New Hampshire, United States of America; 3 Department of Obstetrics and Gynecology and Medicine, Alpert School of Medicine, Brown University, Providence, Rhode Island, United States of America; 4 Department of Pediatrics, Geisel School of Medicine at Dartmouth, Lebanon, New Hampshire, United States of America; 5 Department of Medicine, University of Alabama, Birmingham, Alabama, United States of America; 6 Department of Microbiology, University of Alabama, Birmingham, Alabama, United States of America; 7 Department of Pathology, University of Alabama, Birmingham, Alabama, United States of America; University of Toronto, Canada

## Abstract

**Background:**

We have previously demonstrated intrinsic anti-HIV activity in cervicovaginal lavage (CVL) from HIV-infected women with high CD4 counts and not on antiretroviral therapy. However, the impact of HIV disease progression on CVL innate immune responses has not been delineated.

**Methods:**

CVL from 57 HIV-infected women not on antiretroviral therapy were collected by washing the cervicovaginal area with 10 ml of sterile normal saline. We characterized subject HIV disease progression by CD4 count strata: >500 cells/µl, 200–500 cells/µl, or <200 cells/µl of blood. To assess CVL anti-HIV activity, we incubated TZM-bl cells with HIV plus or minus CVL. Antimicrobials, cytokines, chemokines and anti-gp160 HIV IgG antibodies were measured by ELISA and Luminex.

**Results:**

CVL exhibited broad anti-HIV activity against multiple laboratory-adapted and transmitted/founder (T/F) viruses, with anti-HIV activity ranging from 0 to 100% showing wide variation between viral strains. Although there was broad CVL inhibition of most both laboratory-adapted and T/F virus strains, there was practically no inhibition of T/F strain RHPA.c, which was isolated from a woman newly infected via heterosexual intercourse. HIV disease progression, measured by declining CD4 T cell counts, resulted in a selective reduction in intrinsic anti-HIV activity in CVL that paralleled CVL decreases in human beta-defensin 2 and increases in Elafin and secretory leukocyte protease inhibitor. HIV disease progress predicted decreased CVL anti-HIV activity against both laboratory-adapted and T/F strains of HIV. Anti-HIV activity exhibited close associations with CVL levels of fourteen cytokines and chemokines.

**Conclusions:**

Amid a multifaceted immune defense against HIV-1 and other sexually transmitted pathogens, HIV disease progression is associated with selective disturbances in both CVL anti-HIV activity and specific innate immune defenses in the human female reproductive tract (FRT). Overall, these studies indicate that innate immune protection in the FRT is compromised as women progress to AIDS.

## Introduction

Despite the proven efficacy of condoms and antiretroviral therapy in preventing HIV transmission [1], [2], [3], the continued expansion of the worldwide HIV pandemic is driven predominantly by heterosexual HIV transmission [4]. The development of new ways to prevent heterosexual HIV transmission thus remains a global public health priority. A major obstacle to the development of new approaches to preventing heterosexual transmission of HIV is uncertainty regarding the mechanisms of innate immune protection from heterosexual transmission of HIV infection.

The FRT is home to a robust and interconnected network of mucosal immune defenses that protect against the transmission of HIV and other sexually transmitted infections [5]. In addition to a protective layer of cervical mucous and an epithelial cell lining, multiple immune cell types exist in the tissues of the FRT including macrophages, dendritic cells, natural killer cells and neutrophils [6]. These cells contribute to innate and antigen-specific immune responses against sexually transmitted pathogens that are distinct from immune responses measured in the gut and in peripheral blood samples [7], [8]. Within the FRT, cytokines, chemokines, endogenous microbicides and antibodies constitute the multifaceted layer of soluble innate immune protection against HIV and other sexually transmitted [8].

We have previously shown that CVL samples from HIV(-) and HIV-infected women with CD4+T cell counts greater than 500 have profound anti-HIV activity against a range of laboratory-adapted viruses, as well as against transmitted/founder viruses [9]. We have also shown that CVL from HIV-infected women contain multiple endogenous microbicides such as human beta defensin 2 (HBD2), Elafin, macrophage inflammatory protein-3 alpha (MIP-3α) and secretory leukocyte protease inhibitor (SLPI) along with HIV-specific IgG. As part of these studies, we found a correlation between anti-HIV activity of CVL from HIV(−) and HIV-infected women with HBD2 and MIP-3α as well as the levels of CVL IgG directed at HIV gp160 [9]. However, the exact functional contribution of these CVL immune responses remains unclear, as does the impact of HIV disease progression on each of these likely overlapping and interacting immune responses.

HIV disease progression is associated with generalized immunodeficiency [10], chronic immune activation [11], [12], deterioration of HIV-specific T cell responses [13], [14], and higher circulating HIV viral loads [15]. It is plausible therefore that HIV disease progression results in damage to the innate and adaptive immune responses in the FRT. Yet, although CVL HIV viral load increases with HIV disease progression [16], the impact of HIV disease progression on CVL innate immune responses and anti-HIV activity is not known. Given that dysregulation and/or loss of immune responses in the genital tract has the potential to make women more prone to transmitting HIV, it is critical to define the impact of HIV disease progression on innate immune responses in the CVL.

We hypothesized that HIV disease progression attenuates both the anti-HIV activity and protective immune responses within the FRT. To address this hypothesis, we assessed the impact of HIV disease progression on the anti-HIV activity and innate immune responses of CVL from HIV-infected women.

## Methods

### Subject Recruitment

The 57 HIV-infected and sexually-abstinent women in this study were recruited from The Immunology Center, Miriam Hospital as a part of an observational study on HIV shedding in women. CVL from 15 HIV-uninfected women were obtained from the Rhode Island site (Miriam Hospital, Brown University, Providence, RI) of the HIV Epidemiology Research (HER) study. Studies were conducted according to the principles expressed in the Declaration of Helsinki and was approved by the Miriam Hospital Institutional Review Board (Brown University, Providence, RI) as well as the Dartmouth College Committee for the Protection of Human Subjects (Hanover, NH). All patients provided written informed consent for the collection of samples and subsequent analysis.

### HIV-infected Study Participants

Women were aged 38–49 years. Enrollment criteria included a normal menstrual history, not on hormonal contraceptives, and no exposure to antiretroviral (ARV) drugs. For disease progression analyses, patients were categorized into three groups based on CD4 T cell counts: >500 cells/µl, 200–500 cells/µl, and <200 cells/µl. Participants agreed to undergo gynecologic assessment, and were excluded for pregnancy, breastfeeding, menopause, or inter-menstrual bleeding in the previous three months. Additionally, women were excluded if they had douched, used any vaginal products, or had sexual intercourse during the 48 hrs prior to CVL collection. CVL was collected by gently washing the cervicovaginal area with 10 ml of sterile normal saline (pH∼7.2). Following CVL collection, samples were centrifuged at 10,000×g for 5 min after which supernatants and cell pellets were stored at −80°C until used. Women were tested for lower genital tract infections including but not limited to bacterial vaginosis (BV), *Trichomonas vaginalis, Neisseria gonorrhea* and *Candida albicans*.

### HIV-uninfected Participants

Women were aged 24–34 years. CVL were collected using an identical approach to that described above. Following CVL collection, samples were immediately frozen at −80°C. At the time of analysis, samples were thawed to room temperature, centrifuged at 10,000×g for 5 min after which supernatants were assayed for anti-HIV activity. In preliminary studies (not shown), we compared CVL that were centrifuged after collection and prior to freezing with those that were freeze-thawed once prior to centrifugation and found no differences in anti-HIV activity.

### HIV Viral Stocks

Laboratory-adapted viral strains HIV-1 IIIB (X4) and BaL (R5) were obtained from Dr. P. Gupta (University of Pittsburgh, PA). Virus stocks produced in PBMC of molecularly cloned HIV-1 NL4.3 (X4; laboratory-adapted) and YU-2.c (R5; directly cloned from brain tissue without culture were also used in this study [17]). Also used was a PBMC-derived virus stock of CH077.c, CH058.c, and RHPA.c, all sexually transmitted Clade B infectious molecular clones (IMC) [18], [19], [20] matching the inferred T/F virus nucleotide sequence from subjects 700010077, 700010058, and RHPA4259 respectively. The RHPA.c is currently only available full-length clade T/F IMC isolated from a heterosexually-infected female subject. Virus stocks were propagated in PHA-stimulated human PBMC and stored frozen at −80°C. Virus titers were determined on TZM-bl cells as described [21].

### Measurement of CVL Anti-HIV-1 Activity

Intrinsic anti-HIV activity in CVL was determined using TZM-bl cells. The TZM-bl indicator cell line is a HeLa cell derivative that expresses high levels of CD4, CCR5 and CXCR4 as well as an HIV long terminal repeat (LTR)-driven β-galactosidase gene plus firefly luciferase reporter cassettes that are activated by HIV Tat protein expression and whose use has been standardized for the measurement of HIV-1 infectivity by co-author J.C.K. [22], [23]. TZM-bl cells were routinely sub-cultured every 3 to 4 days by trypsinization and maintained in TZM-bl media consisting of phenol red-free DMEM (Invitrogen Life Technologies, Carlsbad, CA) supplemented with 10% defined FBS (HyClone, Logan, UT), 2 mM L-glutamine (Invitrogen Life Technologies), and 50 µg/ml primocin (Invivogen, San Diego, CA).

TZM-bl cells were seeded at 2×10^4^ cells per well in a 96-well microtiter plate and allowed to adhere overnight at 37°C. CVL from individual patients were diluted 1∶4 in TZM-bl media and incubated with virus (MOI = 1, as determined on TZM-bl cells) for 1 hr at 37°C in a final volume of 100 µl. Following incubation, media were aspirated from TZM-bl cells and the virus plus CVL mixture (100 µl) was added to the cells along with 100 µl of TZM-bl media. Luciferase activity was measured on a luminometer after application of beta-Glo (Promega Corporation, Madison, WI) per manufacturer’s instructions. Controls included incubation of TZM-bl with virus alone, CVL alone and cells in media. Uninfected cells and cells treated with CVL alone were used to determine background luminescence and data expressed in relative light units (RLU). To calculate percent inhibition, the RLU values of “virus only” wells were averaged and set to 100%. Values of CVL treated virus were calculated as a percentage of the “virus only” and then subtracted from 100 to calculate % inhibition. Viability of TZM-bl cells upon treatment with CVL was quantified using the CellTiter 96® AQueous One Solution Cell Proliferation Assay (Promega) according to manufacturer’s instructions. Briefly, reagent was added directly to cell cultures and incubated for 30 min at 37°C followed by reading the plate in a plate reader at OD 490 nm.

### Measurement of Cytokines, Chemokines, Antimicrobials in CVL

CVL supernatants were stored at –80°C until assayed for SLPI, MIP-3α and Elafin with ELISA Quantikine kits or ELISA Duoset kit from R&D Systems (Minneapolis, MN) according to the manufacturer’s protocol. Standards for each ELISA were re-suspended in phosphate buffered saline (PBS). CVL samples were also diluted in 1×PBS. Cytokines were quantified based on standard curves obtained using an ELISA reader (Dynex, Chantilly, VA). HBD2 was assayed with an ELISA test kit from PeproTech (Rocky Hill, NJ) according to the manufacturer’s protocol. For LUMINEX analyses, CVL were assayed for 14 different chemokines and cytokines (BioRad, Hercules, CA), as previously described [24].

### Measurement of Anti-gp160 HIV IgG Antibody Levels in CVL

Specimens were tested by kinetic ELISA (kELISA) adapted from an assay previously described for influenza [25]. The kELISA measures substrate activation every 9 sec over the first 5 min of the enzymatic assay and plots the change in color per min as mOD/min using a Thermomax microplate reader (Molecular Devices, Sunnyvale, CA). Duplicate wells were coated with MN strain gp160 (Protein Sciences, Meriden, CT). An uncoated well was run with each sample to determine background, which was subtracted from the result. A standard curve with a known positive serum specimen was included in each assay as a measure of sensitivity and reproducibility. Biotinylated anti-human IgG conjugates were used with streptavidin-HRP and ABTS [25]. The presence of anti-HIV gp160 specific IgG antibodies used 15 m OD as a cut-off for detection.

### Measurement of HIV-1 RNA in CVL and Plasma

Nucleic acid sequence-based amplification (BioMerieux, Durham, NC) was used to measure HIV-1 RNA as previously described [26]. Briefly, we used a commercially available nucleic acid sequence-based amplification assay (NASBA) technique for quantification of HIV-1 RNA in paired plasma and cervicovaginal lavage samples (Organon Teknika Corp., Durham, NC). This method is isothermal and amplifies only HIV-1 RNA and not proviral DNA. Blood specimens were anti-coagulated in ethylenediamine tetraacetic acid, and plasma was separated within 1 hr of collection. An aliquot of 200 µl or 1 ml of unfractionated cervicovaginal lavage was used for HIV-1 RNA quantification. The plasma HIV-1 RNA viral load was measured using 100 µl or 1 ml of plasma. Cervicovaginal lavage and plasma were added to lysis buffer within 3 hr of collection and stored at −70°C. Specimens were shipped in dry ice to Massachusetts General Hospital, and stored at −70°C until they were run for HIV-1 RNA testing. All results are expressed as copies per ml.

### Statistical Analysis

We contrasted CVL anti-HIV activity between CD4 count strata using Kruskal-Wallis tests, and conducted two-group comparisons using Mann-Whitney *U* tests, in both cases using a P value significance threshold of less than 0.05. We measured the association between CD4 count, PVL and GTVL with CVL anti-HIV activity as well as CVL cytokine, chemokine and microbicide levels using Spearman correlation coefficients with a P value threshold for statistical significance of 0.01 to adjust for multiple comparisons. We also assessed the correlation between GTVL and anti-HIV activity as well as CVL microbicide and chemokine levels using linear regression while adjusting for the PVL. We conducted data analyses using GraphPad Prism (La Jolla, CA) and STATA 9 (College Station, TX).

## Results

### Subject Demographic Characteristics

The 57 HIV-infected subjects not on antiretroviral therapy that provided CVL samples for this study had diverse CD4 counts (median 510 cells/µl, range 19–1,517 cells/µl), peripheral HIV viral loads (median 4,650, range <200–650,000 copies/ml) and CVL HIV viral loads (median 400, range <200–1,575,000 copies/ml). There were 30 subjects with CD4 counts >500 cells/µl, 21 subjects with CD4 counts 200–500 cells/µl, and 6 subjects with CD4 counts <200 cells/µl.

### CVL from HIV-infected Women have Variable Anti-HIV Activity Against X4 and R5-tropic HIV-1 Including Transmitted/Founder (T/F) HIV-1 Strains

**Figure 1:**
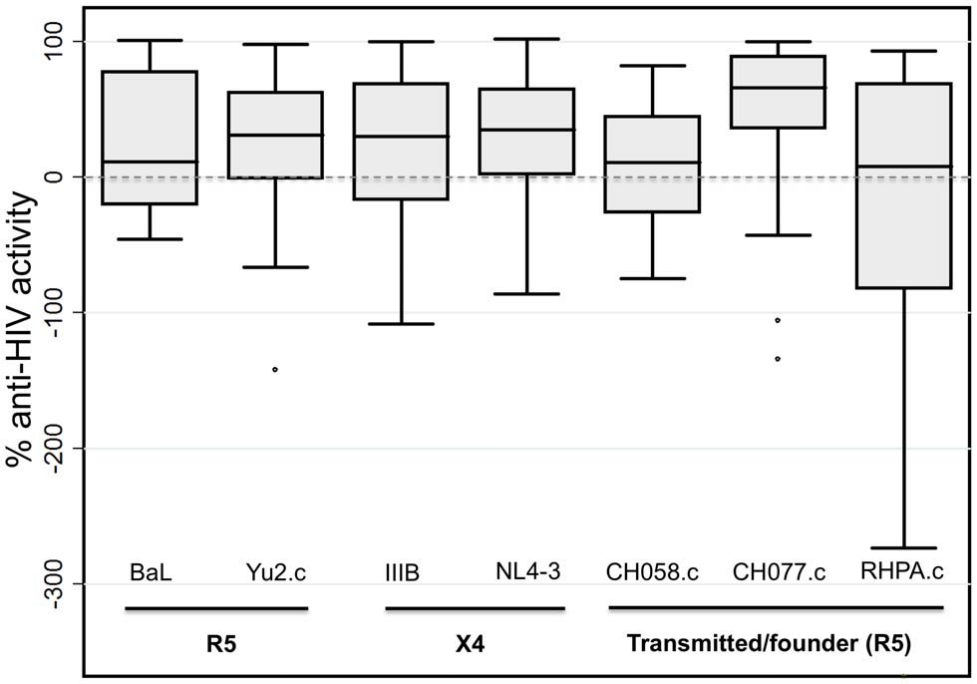
Broad and highly variable anti-HIV-1 activity of cervicovaginal lavage fluid from 57 HIV-infected women against multiple R5 and X4 strains of HIV-1, including transmitted/founder viruses. Bars depict median, interquartile range and 95% confidence intervals. Negative values indicate enhancement of infection.

To determine whether CVL collected from 57 HIV-infected women inhibit HIV infection of target cells, CVL were incubated with either X4- or R5- tropic HIV-1 prior to the measurement of HIV-1 infectivity via the TZM-bl assay. We used seven different HIV-1 strains including two X4-tropic viruses (IIIB and NL4.3), two R5-tropic/macrophage-tropic viruses (BaL and Yu2.c) and three mucosally transmitted clade B T/F viruses, CH058.c, CH077.c and RHPA.c, which can utilize CCR5 co-receptor [20]. We observed that the median values of anti-HIV activity of CVL samples varied widely (from 368% enhancement to complete inhibition) (Figure 1). Furthermore, among individual subjects we found a broad spectrum of anti-HIV activity against any given HIV-1 strain ranging from 100% inhibition of viral replication to enhancement (<0% inhibition) of viral infectivity of target cells.

Although T/F strain CH077.c and CH058.c were inhibited comparably to the other reference strains, heterosexually-transmitted RHPA.c was not as inhibited and also showed the greatest propensity for enhancement of infection in the presence of CVL. Overall, no single CVL sample showed clear inhibition of all viral strains. Further, some CVL samples with substantial activity against one HIV-1 strain exhibited substantially less activity against a different HIV-1 strain, and there was no clear pattern of CVL activity against pairs of virus strains or against one viral tropism over another. Our data show that a broad spectrum of HIV inhibitory activity against multiple strains exists in the CVL of our cohort of HIV-infected women.

### HIV Disease Progression Results in a Significant and Selective Reduction of Intrinsic Anti-HIV Activity in CVL

**Figure 2:**
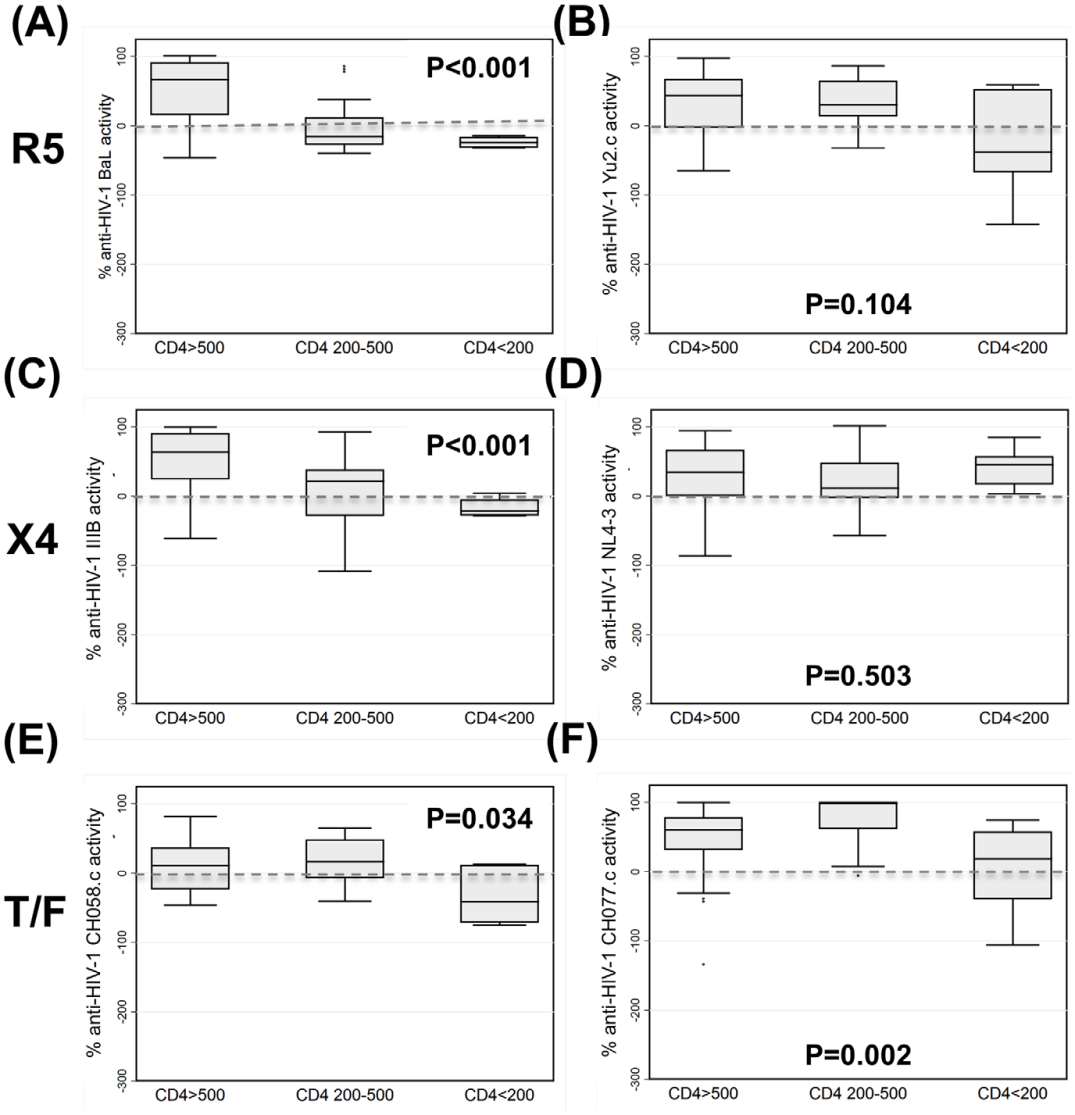
HIV disease progression exerts a selective impact on anti-HIV activity of cervicovaginal lavage fluid. HIV disease progression to CD4 counts <200 was associated with significant reductions in anti-HIV activity against the R5-tropic virus BaL (A) but not Yu2.c (B), on one of two X4 tropic viruses IIIB and NL4.3 (C &D), and against both transmitted/founder (T/F) viruses CH058.c or CH077.c (E & F). Negative anti-HIV activity values indicate enhancement of infection. Bars depict median, interquartile range and 95% confidence intervals; P values derived via Kruskal-Wallis tests.

We have shown previously that CVL from HIV-infected women with CD4 counts >350 cells/µl exhibit intrinsic anti-HIV activity [9]. In the current study we included HIV-infected women with a broader CD4 count spectrum in the absence of antiretroviral treatment: 30 had CD4>500 cells/µl, 21 had CD4 200–500 cells/µl and 6 had CD4<200 cells/µl. As seen in Figure 2, we found a progressive loss of median anti-HIV activity among women with lower CD4 counts against R5 tropic HIV-1 BaL, X4-tropic HIV-1 IIIB, and against the T/F strains CH058.c and CH077.c. There was no significant loss of median CVL anti-HIV activity for R5-tropic Yu2.c, and X4-tropic NL4-3. Overall our data indicate that HIV disease progression is associated with the loss of CVL anti-HIV activity against multiple HIV-1 strains.

### Anti-HIV Activity in CVL does not Efficiently Inhibit RHPA.c, a T/F Strain Derived from a Female Subject with Heterosexual Transmission Risk

**Figure 3:**
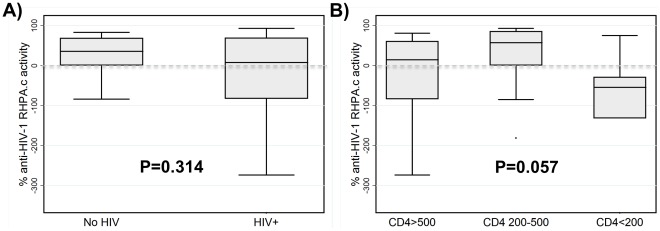
Inhibition by cervicovaginal lavage of T/F virus RPHA.c derived from a woman who reported heterosexual intercourse as her risk for contracting HIV. (A) There was no statistically significant difference between median values of inhibition of RPHA.c comparing women with and without HIV infection. (B) Among HIV-infected women, HIV disease progression stage modulated the inhibitory effect of cervicovaginal lavage fluid on RPHA.c. Negative anti-HIV activity values indicate enhancement of infection. Bars depict median, interquartile range and 95% confidence intervals; P values via (A) Mann-Whitney *U* test and (B) Kruskal-Wallis test.

We recently reported the derivation of the T/F virus nucleotide sequence from subject RHPA4259, and the generation of an infectious molecular clone (IMC) RHPA.c [20]. RHPA.c represents the variant that established clinical infection in this female subject who reported heterosexual transmission as her only risk factor. In current study, we measured anti-HIV activity of CVL from HIV-uninfected as well as HIV-infected women. Of the 15 HIV-uninfected samples, 12 could be tested for activity against RHPA.c. As seen in Figure 3A, the majority inhibited this virus with the exception of three samples, which showed a stimulatory effect. Median percent inhibition in HIV-uninfected women was 36% (range −84% to 83%) whereas in 57 HIV-infected women, the median percent inhibition was 8% (range −368% to 93%; Figure 3A). There was no significant difference in CVL anti-HIV activity against RHPA.c between HIV-infected and HIV(−) women as determined by a Mann-Whitney *U* test. Among HIV-infected women, there was a trend toward lower CVL anti-RPHA.c activity among women with CD4 counts <200 cells/µl compared to women with higher CD4 counts (Figure 3B). In fact, of the 57 HIV-infected women, CVL from 19 (33%) enhanced RHPA.c infectivity of TZM-bl target cells.

### Association Between Peripheral HIV Viral Load (PVL) and Genital Tract HIV Viral Load (GTVL) with Anti-HIV Activity and Immune Parameters in the FRT

Recognizing that PVL is a key predictor of HIV disease progression [16] and HIV transmission [27], and that GTVL is an important determinant of viral shedding and secondary transmission in sero-discordant couples [28], [29], we evaluated the relationship between both PVL and GTVL and anti-HIV activity (Table 1). The PVL correlated negatively with the magnitude of anti-HIV activity against laboratory-adapted HIV-1 BaL and IIIB, while there was no correlation between GTVL and CVL anti-HIV activity either in univariate correlations or in multivariate linear regression adjusting for the PVL. There were no correlations between anti-HIV activity against laboratory-adapted X4 strain NL4.3, R5 strain Yu2.c as well as R5 T/F strains CH077.c, CH058.c, and RHPA.c and any of the clinical parameters (CD4 counts, blood/CVL viral load) measured (Table 1). The GTVL, however, was higher among subjects with lower CD4 counts (Spearman r −0.324, P = 0.015) and correlated closely but imperfectly with the PVL (Spearman r 0.3739, P = 0.0049).

**Table 1:** Correlations between CD4 count, peripheral HIV viral load and cervicovaginal HIV viral load with anti-HIV activity of cervicovaginal lavage from HIV-infected women.

Viruses tested for anti-HIV activity			CD4 count		Peripheral HIV viral load		Cervicovaginal lavage HIV viral load	
Viral Tropism	Virus	N	Spearman ρ	P value	Spearman ρ	P value	Spearman ρ	P value
R5	BaL	57	0.647	<0.001	−0.347	0.009	−0.178	0.189
	Yu2.c	56	0.230	0.088	−0.074	0.591	0.028	0.839
X4	IIIB	57	0.549	<0.001	−0.313	0.019	−0.134	0.326
	NL4-3	45	0.120	0.433	−0.262	0.085	0.251	0.101
(T/F)	CH058.c	51	0.245	0.083	−0.033	0.823	−0.052	0.718
	Ch077.c	56	−0.087	0.523	0.059	0.670	0.153	0.266
	RHPA.c	47	0.021	0.888	−0.130	0.389	0.043	0.778

CVL levels of the laboratory-adapted HIV viruses IIIB and BaL correlated positively with CD4 count and negatively with the peripheral HIV viral load. By contrast, there were no significant correlations of CVL HIV viral load with anti-HIV activity against any virus.

### Predictors of the Levels of Endogenous Anti-HIV Microbicides in the FRT

Previously, we and others demonstrated the presence of endogenous innate anti-HIV factors in CVL [9], [30], [31], [32] and found that the levels of some antimicrobials correlated with anti-HIV activity in healthy HIV-infected women [9]. We confirmed these findings in the present study (data not shown). In the present study, additionally, we measured HBD2, MIP-3α, SLPI, Elafin and anti-HIV gp160 IgG in the CVL of HIV-infected women, and evaluated correlations between levels of antimicrobials and subject CD4 count, PVL, and GTVL (Tables 1 and 2). We found no correlation between GTVL and CVL anti-HIV activity in unadjusted analyses or when analyses were adjusted for the PVL, but we did find significant positive correlations between CVL levels of Elafin and SLPI and both the PVL and the GTVL suggesting that some combination of immunodeficiency and mucosal immune activation contribute to the elaboration of innate mucosal defenses. The sole correlation between GTVL and the 14 measured cytokines/chemokines was with IL-1α (Spearman r 0.410, P = 0.003), although this correlation was not evident in unadjusted linear regression (correlation coefficient −0.002, P = 0.686) or linear regression adjusted for the PVL (correlation coefficient −0.002, P = 0.723).

**Table 2:** Correlations between CD4 count, peripheral HIV viral load and cervicovaginal lavage HIV viral load with the levels of putative anti-HIV microbicides in the cervicovaginal lavage of HIV-infected women.

		CD4 count		Peripheral HIV viral load		Cervicovaginal lavage HIV viral load	
Putative anti-HIV microbicide (pg/ml)	N	Spearman ρ	P value	Spearman ρ	P value	Spearman ρ	P value
**HBD2**	56	0.046	0.732	−0.003	0.984	0.226	0.094
**Elafin**	56	−0.685	<0.001	0.233	0.084	0.336	0.011
**MIP-3**α	56	0.232	0.082	−0.128	0.347	−0.012	0.931
**SLPI**	56	−0.572	<0.001	0.279	0.037	0.326	0.014
**Protein**	51	0.140	0.321	−0.046	0.749	0.248	0.080
**Anti-HIV gp160 IgG**	41	0.215	0.177	−0.035	0.829	0.174	0.283
**Anti-HIV gp 160 IgG, normalized to CVL protein**	40	−0.036	0.826	0.035	0.835	0.088	0.595

CVL levels of the putative endogenous anti-HIV microbicides Elafin and SLPI correlated negatively with the CD4 count, and positively with the CVL HIV viral load. By contrast, the peripheral HIV viral load correlated positively only with CVL levels of SLPI and anti-HIV gp160 IgG.

HBD2, human beta defensin 2; Ig, immunoglobulin G; SLPI, secretory leukocyte protease inhibitor.

### HIV Disease Progression Exerts a Selective Impact on Some Microbicides in CVL Fluid

**Figure 4:**
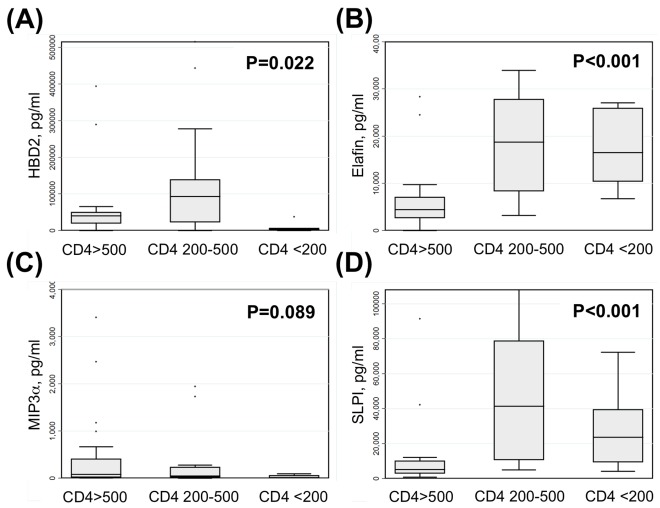
HIV disease progression exerts a selective impact on putative anti-HIV microbicides in cervicovaginal lavage fluid. (A) CVL levels of human beta defensin 2 (HBD2) were significantly lower among women with CD4<200 cells/µl. (B) CVL levels of Elafin were significantly higher among women with CD4<200 cells/µl. (C) There was a trend toward lower CVL levels of macrophage inflammatory protein-3 alpha (MIP-3α) among women with CD4<200 cells/µl. (D) CVL levels of secretory leukocyte protease inhibitor were higher among women with CD4<200 cells/µl. Bars depict median, interquartile range and 95% confidence intervals; P values derived via Kruskal-Wallis tests.

Beyond identifying a correlation between the loss of anti-HIV activity and antimicrobials in CVL and HIV disease progression, we also found a significant and selective impact of HIV disease progression on CVL microbicide levels (Figure 4). Lower CD4 counts were associated with lower CVL levels of HBD2 and a trend toward lower CVL levels of MIP-3α. In contrast, lower CD4 cell counts were associated with higher levels of Elafin and SLPI in CVL. These data suggest HIV disease progress results in a profound but selective impairment in the multifaceted CVL microbicide defense that parallels the selective loss of CVL activity against multiple HIV-1 viral strains seen in Figure 1.

### Predictors of Human CVL Cytokine and Chemokine Responses and Anti-HIV Activity in HIV-Infected Women

To determine whether cytokines and chemokines present in the CVL of HIV-infected women correlate with CD4, PVL, or GTVL, we performed a 14-plex Luminex assay on CVL samples. There were borderline significant correlations of the CD4 count with the levels of two cytokines: MIP-1α (Spearman r 0.293, P = 0.039) and G-CSF (Spearman r 0.2822, P = 0.047). We found that the levels of only 3 effector molecules correlated weakly and negatively with the peripheral HIV viral load: MIP-1α (CCL2; Spearman r −0.318, P = 0.028), MIP-1β (CCL3; Spearman r −0.321; P = 0.025) and TNF-α (Spearman r −0.297, P = 0.038) whereas the majority of chemokines/cytokines exhibited no correlation (data not shown). Of the 14 cytokines/chemokines, 13 correlated significantly with inhibition of at least 1 of the 7 viral strains used for this study (Table 3). When correlations between 14 cytokines/chemokine with CVL anti-HIV activity were adjusted for PVL, only the correlation between MIP-1α and anti-HIV-1 Yu2.c activity became non-significant whereas all other correlations trivially affected (data not shown). By contrast, the significance of a subset of correlations between CVL cytokine/chemokine levels and anti-HIV activity was altered after adjustment for GTVL, suggesting that the relationships of cytokines/chemokine levels to anti-HIV activity are impacted in some instances by mucosal but not systemic virus. Specifically, after adjustment for GTVL, linear regression correlation coefficients (CC) and P values, respectively, were: IL-1RA and RHPA.c, CC 335.71, P = 0.009; GM-CSF and IIIB, CC 0.20, P = 0.007; GM-CSF and Yu2.c, CC 0.26, P = 0.002; GM-CSF and CH058.c, CC 0.41, P<0.001; G-CSF and IIIB, non-significant (NS); G-CSF and BaL, NS; RANTES and all anti-HIV activity, NS. The heterosexually transmitted female T/F virus RHPA.c correlated only with levels of IP-10. Interestingly, IL-1α did not show any correlations with anti-HIV activity against any of the viral strains but the sole correlation between GTVL and the 14 measured cytokines/chemokines was with IL-1α (Spearman r 0.410, P = 0.003). Overall, these data suggest HIV disease progression does not exert a major impact on the constitutive and multifaceted cytokine and chemokine response in the FRT.

**Table 3:** Analysis of associations between the levels of cytokines and chemokines (CK/CC) with anti-HIV activity of the cervicovaginal lavage (CVL) from HIV-infected women against the indicated viral strains.

Cytokine/chemokine	IIIB	NL4.3	Yu2.c	BaL	CH077.c	CH058.c	RHPA.c
IL-1α	NS	NS	NS	NS	NS	NS	NS
IL-1RA	0.374, 0.008	0.414, 0.007	0.463, <0.001	NS	0.390, 0.005	0.566, <0.001	NS*
IL-6	0.438, 0.002	NS	0.433, 0.002	0.511, <0.001	NS	0.524, <0.001	NS
IL-8	NS	0.498, <0.001	NS	0.376, 0.007	0.365, 0.009	0.405, 0.004	NS
IP-10	0.606, <0.001	NS	0.502, <0.001	0.463, <0.001	NS	0.631, <0.001	0.524, 0.007
MCP-1 (CCL2)	NS	NS	NS	NS	NS	0.393, 0.005	NS
MIP-1α (CCL3)	0.560, <0.001	NS	0.538, <0.001	0.524, <0.001	NS	0.501, <0.001	NS
MIP-1β (CCL4)	0.448, 0.001	0.411, 0.007	0.387, 0.006	0.444, 0.001	NS	0.438, 0.002	NS
TNF-α	0.475, <0.001	0.543, <0.001	0.496, <0.001	0.397, 0.004	NS	0.457, <0.001	NS
GM-CSF	NS*	NS	NS*	NS	NS	NS*	NS
G-CSF	0.456, <0.001*	NS	NS	0.526, <0.001*	NS	0.420, 0.002	NS
RANTES (CCL5)	0.556, <0.001*	NS	0.501, <0.001*	0.549, <0.001*	NS	0.563, <0.001*	NS
Eotaxin	0.548, <0.001	NS	0.456, <0.001	0.419, 0.003	NS	0.458, <0.001	NS
Fractalkine (CX3CL1)	0.495, <0.001	0.488, 0.001	0.545, <0.001	0.454, <0.001	0.367, 0.009	0.537, <0.001	NS

Numbers in cells indicate r value for Spearman correlation and P value, unless P value for correlation is >0.01 in which case correlation is listed as not significant (NS). *Significance of correlation altered by adjustment for GTVL (see text for details).

## Discussion

The development of novel immunological means for preventing the sexual transmission of HIV-1 is hindered by continuing uncertainty regarding the critical mechanisms of immune defense against HIV-1 transmission. Here, we show that CVL exhibits both potent anti-HIV immune activity and a rich and multifaceted content of cytokines, chemokines and endogenous microbicides, with extensive correlation between the two and a clear and selective impact of HIV disease progression on both. As a part of these studies we found that HIV disease progression results in a significant and selective reduction in intrinsic anti-HIV activity in CVL, and that this loss of CVL anti-HIV activity is paralleled by significant and selective disturbances in the CVL microbicide content. Overall, these studies indicate that innate immune protection in the FRT is compromised as women progress to AIDS.

Although CVL exhibits broad anti-HIV activity against R5, X4 and T/F viruses [9], HIV disease progression impacts anti-HIV activity of CVL differently for different viral strains. Specifically, CVL anti-HIV activity against the laboratory-adapted isolate IIIB and BaL as well as the sexually transmitted T/F viruses CH058.c, CH077.c, and RHPA.c clearly declined with HIV disease progression. No such pattern was observed for Yu2.c. or NL4.3. Our data offer a possible explanation on why HIV-1 transmission risk may increase with disease progression [33], [34] and has profound implications for the evaluation and development of preventive measures that are active at all phases of HIV disease.

HIV disease progression was associated with specific alterations in CVL immune responses. Levels of both Elafin and SLPI correlated negatively with peripheral CD4 counts, suggesting progressive elevations in these local inflammatory mediators through the course of HIV disease progression. Both Elafin and SLPI levels were correlated with PVL and GTVL, suggesting that local and/or systemic antigen burden may be the prime determinant of these non-specific but putatively protective inflammatory responses. Importantly, the levels of all four putative endogenous microbicides assessed – HBD2, Elafin, MIP-3α and SLPI – were altered by HIV disease progression. To the best of our knowledge, this is the first demonstration that innate immune protection in the human FRT is lost as women with HIV-1 infection progress towards AIDS, and that this loss of anti-HIV activity is mirrored by impairments in endogenous microbicides such as HBD2. Other than impacting overall concentrations of microbicides measurable by ELISA, the biological activities of the molecules might be altered such as through protease digestion. Many of the molecules we measured are expressed as precursor proteins that must be cleaved for activation and/or release in secretions by a spectrum of endogenous proteases, which are present in FRT secretions [35], [36]. For example, the cathepsin and kallikrein families of proteases are present in FRT secretions and can directly modulate the biological activities of a number of anti-HIV innate immune factors such as MIP-3α, defensins, and LL37 [37], [38], [39]. Since ELISA assays measure levels but not biological activity of anti-HIV molecules, this could be an explanation for enhanced susceptibility of some women but not others.

The levels of nearly all 14 cytokines and chemokines we measured correlated significantly with CVL anti-HIV activity, but not with HIV disease progression. Notably, no single cytokine or chemokine correlated with anti-HIV activity against all tested viral strains. While these data suggest that the maintenance of robust and multifaceted immune responses are important to innate immune protection against HIV and other sexually transmitted pathogens, they do not identify a single mechanistic explanation for the clear and documented CVL anti-HIV activity. It will be a high priority to test the impact of blocking chemokines and cytokines individually and in combination on CVL anti-HIV activity against an expanded panel of relevant reference and T/F viruses. This approach will allow us to identify which inflammatory mediators are most critical to immune protection from HIV-1 transmission and thus candidates for novel combination topical approaches to transmission reduction.

Systemic and mucosal HIV viral loads were major drivers of CVL levels of the immune mediators Elafin, SLPI and IL-1α. In addition, correlations between a subset of CVL cytokines/chemokines with anti-HIV activity, including those with GM-CSF, G-CSF and RANTES, were altered after adjustment for GTVL but not PVL by linear regression. This suggests but does not prove that some mucosal immune responses are preferentially impacted by the presence of virus at mucosal surfaces. By contrast, the levels of most measured endogenous antimicrobials, cytokines and chemokines were either constitutive or driven by the presence of alternate (non-HIV) pathogens. Given the known association between both sexually transmitted infections and bacterial vaginosis with the risk of sexual transmission of HIV-1 [40], [41], it will be critical to identify the modifiable drivers of the levels of innate immune mediators in CVL and their impact on CVL anti-HIV activity.

By relating the broad anti-HIV activity of CVL with HIV disease progression and multiple innate immune responses, these studies provide a framework for future investigations into the mechanisms of innate immune protection against HIV that can be used in the development of novel approaches to prevent the sexual transmission of HIV-1. Although the immune responses we measured in the CVL likely constitute the first line of immune defenses against HIV-1, other mucosal immune responses also avert HIV-1 transmission and were not assessed in this study. None of our subjects were on antiretroviral therapy, which is essential to determine intrinsic anti-HIV activity of CVL. However, this study design precludes assessment of a different yet important question: the impact of ART on CVL anti-HIV activity or innate immune responses. We hypothesize ART will simultaneously boost anti-HIV activity while reducing the local and systemic inflammatory triggers for the release of putative anti-HIV mediators like Elafin and SLPI.

As we showed previously [9], some CVL samples inhibit, enhance, or have no effect on HIV infection of target cells depending upon the viral strain used. FRT secretions contain a multitude of innate immune mediators that fluctuate with the menstrual cycle [31], [42]. For instance, CVL levels of HBD1, HBD3, HBD4, SLPI, and Elafin all peak during different stages of the cycle [30], [43], [44], [45]. Previously, we found that these changes are due to sex hormones in that SLPI and HBD2 are regulated by estradiol [46], [47]. Thus, one possible explanation for the variability of activity against different viral strains is that subject samples were collected independent of menstrual status. However, in other cases, we found that a given CVL sample with potent activity against one HIV strain had no activity against others. This finding suggests that different molecules in CVL have differential biological activity against different strains of HIV, indicating that the overall anti-HIV activity of a given CVL sample is determined by synergistic or antagonistic interactions between CVL innate immune factors, most likely due to the changing hormonal milieu of the menstrual cycle. Our data reflect only one clade B T/F virus from a female subject who likely acquired HIV through heterosexual transmission. Future studies with a greater variety of T/F viruses, and with an array of R5 and X4 viruses, will be important to determine if T/F viruses from different transmission mechanisms and with different cellular tropisms exhibit differential abilities to elude innate immune protective responses. We suspect it is unlikely there is a single factor responsible for anti-HIV activity in FRT secretions, and rather that it will be most important for the development of novel immunological strategies for the topical prevention of HIV-1 transmission to identify the factors most predominantly and commonly prevent transmission when acting in concert.

In conclusion, our data depict the multifaceted innate immune responses in CVL against HIV-1, and newly establish that these factors are not only selectively impacted by HIV disease progression and only partly impacted by local and systemic HIV infection. Understanding the impact of this multifaceted mucosal defense system against a wide array of HIV-1 strains including T/F viruses will aid the development of novel approaches to the combined topical prevention of HIV-1 transmission. It will be particularly critical to harness key innate immune mediators that contribute to CVL anti-HIV activity despite HIV disease progression.
